# The effect of age, sex and a firm-textured surface on postural control

**DOI:** 10.1007/s00221-021-06063-2

**Published:** 2021-05-14

**Authors:** Francesco Palazzo, Alessandra Nardi, Niloofar Lamouchideli, Alfio Caronti, Anas Alashram, Elvira Padua, Giuseppe Annino

**Affiliations:** 1 School of Human Movement Science, Faculty of Medicine and Surgery, University of Rome ‘‘Tor Vergata’’, Via Giovanna Garzoni 39, 00133 Rome, CAP Italy; 2 Department of Mathematics, University of Rome “Tor Vergata”, Rome, Italy; 3 PhD School of Neuroscience, Faculty of Medicine and Surgery, University of Rome ‘‘Tor Vergata’’, Rome, Italy; 4 Department of Human Sciences and Promotion of the Quality of Life, San Raffaele Roma Open University, Rome, Italy; 5 Department of Medicine Systems, University of Rome ‘‘Tor Vergata’’, Rome, Italy

**Keywords:** Plantar mechanoreceptors, Balance, Texture, Postural control, Age-related, Sex-related, Elderly

## Abstract

In previous studies, the influence of plantar sensation has been examined using various textured surfaces with different stiffness materials to assess static balance. This study investigated the effects of a Firm Textured Surface (FTS) along with age and sex-related influences on postural control under different visual conditions. Forty subjects (20 elderly, 10 males, mean age 68.30, 10 females, mean age 68.00, and 20 young people, 10 males, mean age 25.45, 10 females, mean age 27.30) participated in this study maintained a quiet standing on FTS, foam and firm surfaces with eyes open and closed. The center of pressure displacement (CoP_DISP_), CoP velocity (CoP_VEL_), and sway velocity of the CoP in anteroposterior (AP) and mediolateral (ML) direction (V_A/P_ and V_M/L_) were measured. FTS was associated with lower postural sway measures in both the groups with eyes open and closed. However, the foam surface showed the worst results in all postural parameters under all experimental conditions. Separate four-way ANOVAs were applied to each dependent variable. The main effects of surface (*p* < 0.0001), vision (*p* < 0.0001) and age (*p* < 0.0001 for CoP_DISP,_ CoP_VEL_ and V_A/P_; *p* = 0.0003 for V_M/L_) were significant in each of the four fitted models. Sex was never significant, either as a main effect or an interaction with other experimental factors. Eyes open were able to reduce the negative effects of the foam surfaces but without vision the proprioceptive sensory system cues of the body state become more important for maintaining balance. A good stimulation with rigid texture should be considered as relief to reduce the physiological-related decline of afferent information with age.

## Introduction

The task of maintaining upright standing posture requires information from vision, the vestibular system and plantar tactile sensory inputs (Winter 1995; Mesquita et al. 2015). In previous research has been reported that the efficiency of postural control system depends mostly on the afferent activity from plantar cutaneous mechanoreceptors (Kavounoudias et al. 1998; Nurse and Nigg 2001b; Meyer et al. 2004; Zehr et al. 2014) located in the glabrous skin of human foot sole that is activated only in the presence of pressure, load, vibration (Priplata et al. 2003; Patel et al. 2011; Strzalkowski et al. 2017) and skin stretch sensory stimuli (Kennedy and Inglis 2002). Loss of just one of the three sensory systems can cause deterioration in postural stability. Indeed, vestibular dysfunction and/or lack of vision (Magnusson et al. 1990) cause body sway which increases with age. In this contest, plantar mechanoreceptors play an important role in body balance (Annino et al. 2015; Palazzo et al. 2015). In fact, it has been shown that reducing information from receptors located in skin through cooling foot sole (Nurse and Nigg 2001b), anesthesia (Meyer et al. 2004), ischemia conditions (Horak et al. 1990) and/or eliminating sensory information (Nejc et al. 2010) is associated with an increase in postural sway under perturbed postural responses. Controversy, stimulation of foot sole (Qiu et al. 2012; Wang et al. 2016) could lead to an improvement of balance (Hlavackova and Vuillerme 2012) through the modulation of load of lower limbs and the positioning of feet (Zehr et al. 2014).

Moreover, people over 60 years of age have more difficulties in maintaining upright posture due to their marked functional decline (Prieto et al. 1996; Kitabayashi et al. 2011) that can be related to the nervous system, muscles, joints or other causes (Prieto et al. 1996). Reciprocal relation between vision and mechanoreceptors is considered a critical factor that deteriorates progressively over years (Lord et al. 1991; Fitzpatrick and McCloskey 1994; Lord and Ward 1994). These sensory deficits of lower limb somatosensation can lead to an increase risk of falls (Tinetti et al. 1988; Shumway-Cook et al. 1997). To examine balance control, researchers have examined various features of postural response with different support surfaces positioned underneath foot (Chiang and Wu 1997; Blackburn et al. 2003; Jeka et al. 2004; Vrancken et al. 2005; Fransson et al. 2007; Patel et al. 2008). When standing on a foam surface, information from the cutaneous mechanoreceptors of sole of foot is less reliable (Chiang and Wu 1997; Perry et al. 2000), changing the patterns from receptors, increasing postural sway and changing standing strategy (Nurse and Nigg 2001a; Fransson et al. 2007). In addition, both young and elderly subjects showed differences in postural control, especially under altered sensory conditions such as altered vision (eyes closed) and surface (a soft contact surface). Instead, it would appear that this gap is reduced due to a compensation from remaining sensory sources, even if only one of sensory inputs was excluded or interrupted (Teasdale et al. 1991). However, standing on a textured surface resulting in an improvement of the Center of Pressure (CoP) displacement, which represents the position of the vertical ground reaction forces (Mancini and Horak 2010), CoP velocity mediolateral range (Corbin et al. 2007; Palluel et al. 2008; Hatton et al. 2011; Li et al. 2019) and a decreased co-contraction of agonist and antagonist muscles (Nurse and Nigg 2001b). Several studies have suggested a positive relationship between balance/postural regulation, and somatosensory feedback provided by the use of textured (Hlavackova and Vuillerme 2012; Losa Iglesias et al. 2012), especially in eyes-closed condition (Corbin et al. 2007; Qiu et al. 2012; Kenny et al. 2019a) while others have not found any effects with eyes open (Corbin et al. 2007; Hatton et al. 2009). Controversy, other authors demonstrates that textured surfaces have not always improved postural control (Wilson et al. 2008; Hatton et al. 2012; Qu 2015). In this context, an important variable of textured surfaces or insoles could be related to material stiffness. Some studies found improvements in postural control during static balance using soft textured insoles made in Evalite Pyramid EVA (Kenny et al. 2019b) or rigid textured surface made from plastic floor matting material (Corbin et al. 2007; Annino et al. 2015; Vieira et al. 2017) in healthy young subjects. Other studies used soft (Hatton et al. 2011; de Morais Barbosa et al. 2018), semi-rigid (Palluel et al. 2008), and rigid materials (Palazzo et al. 2015; Annino et al. 2018) to investigate postural stability in elderly people finding different results about analyzed postural parameters. Palluel et al. (2008, 2009) investigated the effects of sandals equipped with spike insoles, finding no immediate effects, but an improvement of postural control after 5 min standing or walking in elderly and young adults. Differently, (Qiu et al. 2012) investigated different stiffness materials, finding a significant and progressive decrease in postural sway from barefoot with the use of hard and soft textured insole surfaces in older group and small improvements in younger participants when standing on textured surfaces. In addition to textured materials, some authors have used different additional thicknesses to improve sensory information from sole of foot (Janin and Toussaint 2005; Janin and Dupui 2009; Viseux et al. 2018), suggesting that depression of material stimulated the units of cutaneous mechanoreceptors of type I and II (Forth and Layne 2007, 2008) thereby improving neuromuscular activity (Viseux et al. 2019). Furthermore, some authors investigated sex, and age effects on postural stability on non-stimulating surfaces, finding conflicting results. Some studies found sex-related differences (Overstall et al. 1978; Kim et al. 2010) differently to other studies (Røgind et al. 2003; Demura et al. 2008) without clarifying whether sex and aging processes related to it affect postural control ability. Considering that most of studies investigated soft and semi-rigid textured surface into age-related effects on balance, the aim of this study was to analyze the effects of Firm Textured Surface (FTS), with no deformable spikes along with age and sex-related influences on postural control in various vision conditions comparing postural sway measures (Center of Pressure displacement, Center of Pressure velocity, and sway velocity in anterior–posterior and mediolateral directions).

## Materials and methods

### Participants

Data from two groups of participants were used in this study. The first group consisted of 20 healthy older people (10 males, mean age 68.60 ± 4.74 years, mean height 173.30 ± 5.91 cm, mean weight 82.60 ± 14.94 kg; and 10 females, mean age 68.00 ± 5.17 years, mean height 160.78 ± 5.49 cm, mean weight 68.33 ± 5.41 kg). The second group included 20 healthy young subjects (10 males, mean age 23.60 ± 2.46 years, mean height 174.10 ± 6.77 cm, mean weight 66.80 ± 6.44 kg; and 10 females, mean age 27.30 ± 3.47 years, mean height 160.1 0 ± 4.79 cm, mean weight 55.90 ± 7.52 kg).

All subjects had normal vision or corrected to normal with glasses, and reported no history of balance deficits, neurologic disorders, or musculoskeletal injury, and signed the informed consent form granted by the Institutional Review Ethics Board prior to the test. More precise information regarding the aims, benefits, and risks was provided to all the participants set by the Helsinki Declaration. All assessments were performed in a controlled laboratory environment for this study.

### Equipment

The center of pressure displacement (CoP_DISP_) was measured using a custom-built force platform with six uniaxial load cells of which three were arranged on the left side and another three on the right side (Posture2000—S.A.I.R. s.r.l., Santa Rufina di Cittaducale, Rieti, Italy), and using a PC, which was connected through an amplifier. Signals from the force platform were sampled at 1 kHz amplified, converting analog signals to digital form. Data were smoothed using low-pass digital filters with a cut-off frequency of 10 Hz. All variables were calculated with Posture2000 software. For each foot, ground reaction forces and CoP measures were recorded from the force platform. Both A/P and M/L CoPs were recorded: the left CoP (CoP*l*) and right CoP (CoP*r*) plus the CoP_DISP_ as calculated from a weighted average of the two CoPs (Winter et al. 1996); CoP_DISP_ = CoP*l* * R*vl* / *(*R*vl* + R*vr*) + CoP*r* * R*vr* / *(*R*vl* + R*vr*), where R*vl* and R*vr* are the left and right vertical reaction force, respectively. The CoP velocity (CoP_VEL_) and sway velocity of CoP in A/P and M/L direction (V_A/P_ and V_M/L_) were measured. Each CoP_DISP_, CoP_VEL_, V_A/P_ and V_M/L_ measurements were performed under the same condition.

### Experimental procedures

The test battery consisted of a bipedal stance on the three different surfaces (firm-textured surface, besides, the foam and firm surface as control surfaces) with eyes open (EO) and eyes closed (EC). Each test duration was 20 s (Le Clair and Riach 1996) to avoid fatigue for a total of 6 tests for each subject. Each registration was begun shortly after the operator's assistance asked to remain motionless. Each CoP_DISP_, CoP_VEL_, V_A/P_ and V_M/L_ were analyzed for all 20 s. There were 120 s of rest time between the different surface tests. The surfaces were used for each participant in a randomized order. Each participant performed the test battery in eyes-open condition and the testing location was free of visual and acoustic distraction. After the eyes-open trial, the subject sat on a chair for approximately two minutes before the procedure was repeated with eyes closed.

**Fig. 1:**
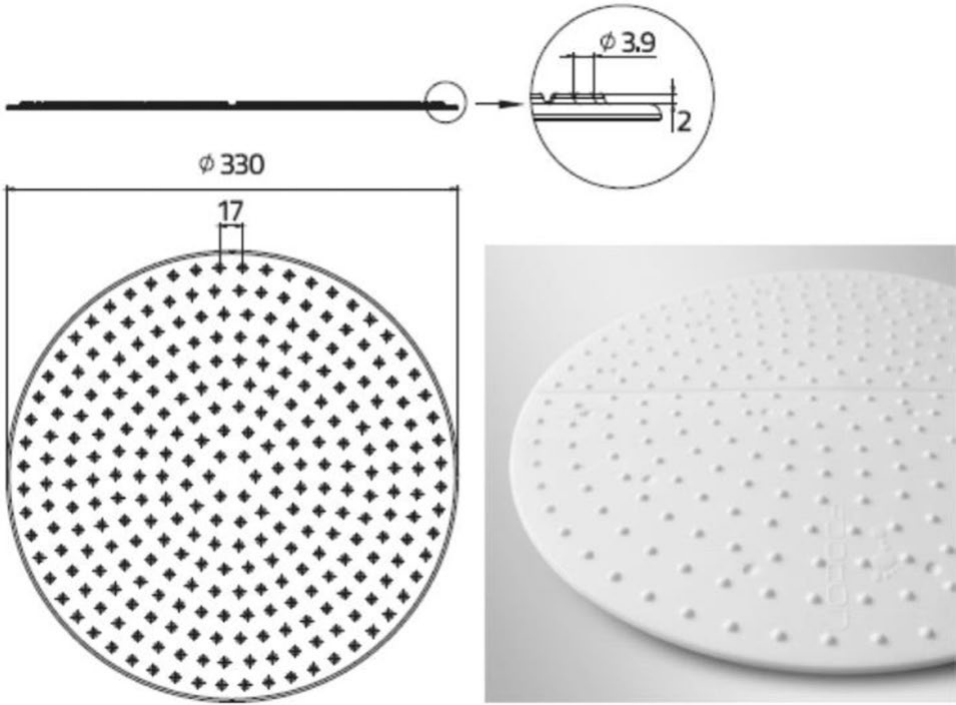
Schematic representation of Firm Textured Surface. Measures are expressed in millimeters

The thickness of the foam mat was 7 mm. In the measurement setup, the foam was placed on top of the force platform without contacting surrounding grounds. The firm-textured disc (Footon-Servetto, Milan, Italy) was 33 cm in diameter with many semi-circular protrusions spaced 17 mm apart, height of 3.5 mm, and width of 2 mm (Fig. 1).

Under the different surface conditions, all subjects stood barefoot with the full length of both feet in contact with the force platform with their arms relaxed along their side to avoid inappropriate results, and they were also asked to sway as little as possible. Both feet were abducted at 30° and the heels were spaced 9 cm apart (Palazzo et al. 2019). Participants were not familiarized with the surfaces before the test.

### Statistical analysis

As the first step in the statistical analysis, a marginal univariate analysis of measured data was performed to determine significant differences among the three considered surfaces in the different conditions. Continuous variables were described by mean and standard deviation. Box and Whisker plots were created to show the distribution of responses among the different surfaces. The Kolmogorov–Smirnov test was used to validate the assumption of normality. Since no significant departures from normality were detected, at multivariable analysis four-way Analysis of Variance (ANOVA) was used to model observed responses in terms of the main effects and possible two-way interactions. Data analysis was limited to two-way interactions to guarantee consistency between the number of parameters to be estimated and the sample size. Four different separate models were fitted, assuming as response variables CoP_DISP_, CoP_VEL_, V_A/P_, and V_M/L_, respectively. In each model, Surface, Vision, Age and Gender were assumed as independent variables; we considered 3 levels for Surface × 2 levels for Vision × 2 levels for Age × 2 levels for Sex. The F-test was used to detect significant effects, assuming α = 0.05. The correlation among measurements from the same participant was modeled by introducing a variance and covariance matrix whose structure was unspecified completely. For significant effects, estimated differences were reported and evaluated using the *t*-test. The Tukey–Kramer adjustment for multiplicity was used. All analyses were undertaken using SAS version 9.4 (SAS Institute, Cary NC).

## Results

**Fig. 2:**
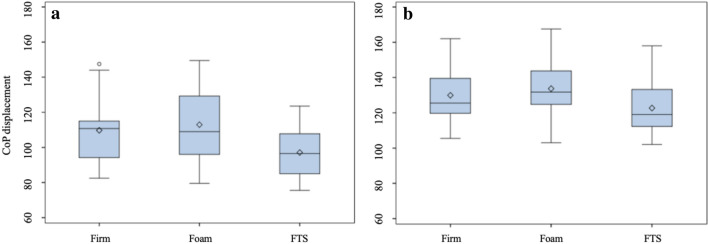
Distribution of CoP displacement for different surfaces with no vision condition. The bottom and top edges of the box are located at the 25th and 75th percentiles of the sample and, within the box, the median is displayed as a line and the mean as a diamond, vertical lines end at the largest and the smallest observed value unless outlying observations (circles) are present. (a) Young individuals, (b) Elder individuals

**Fig. 3:**
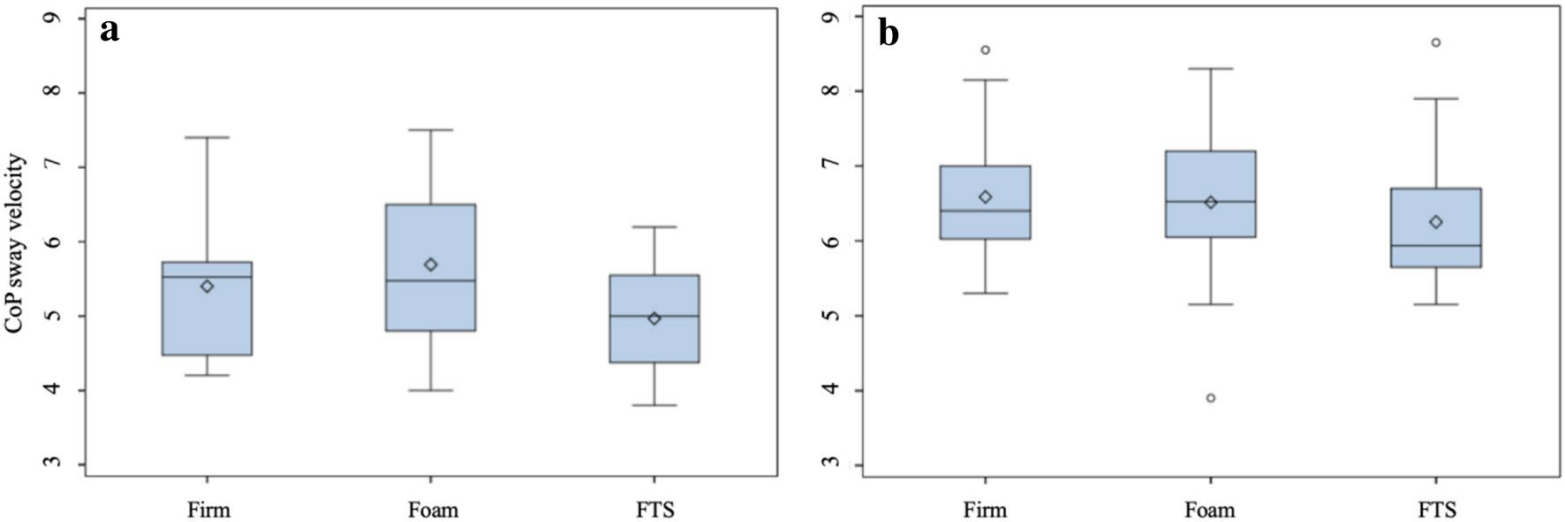
Distribution of CoP sway velocity for different surfaces with no vision condition. The bottom and top edges of the box are located at the 25th and 75th percentiles of the sample and, within the box, the median is displayed as a line and the mean as a diamond, vertical lines end at the largest and the smallest observed value unless outlying observations (circles) are present. (a) Young individuals, (b) Elder individuals

The first descriptive analysis (Table 1) showed that the elderly was significantly unstable in all the analyzed postural parameters than the young people. The differences were more pronounced in the eyes-closed condition. In the eyes-open condition, the gap between the young and old individuals was reduced when standing on the foam surface. In the comparison among the different surfaces, FTS was associated with lower postural sway measures in both the groups with open and closed eyes (Figs. 2 and 3). On the contrary, the foam showed the worst results in all postural parameters during all the experimental conditions.

**Table 1:** Postural parameters mean (mm) and standard deviation (SD)

	Eyes open			Eyes closed		
	Firm	Foam	FTS	Firm	Foam	FTS
CoPDISP (mm)						
Young	98.50 ± 14.29	103.70 ± 15.03	92.48 ± 15.64	109.70 ± 19.21	112.93 ± 19.77	97.08 ± 15.12
Elderly	110.70 ± 15.15	113.00 ± 16.34	104.40 ± 14.27	129.90 ± 15.94	133.70 ± 16.56	122.70 ± 15.84
CoPVEL (mm/s)						
Young	4.86 ± 0.64	5.16 ± 0.74	4.66 ± 0.79	5.40 ± 0.95	5.69 ± 1.03	4.97 ± 0.73
Elderly	5.55 ± 0.70	5.67 ± 0.80	5.26 ± 0.68	6.59 ± 0.89	6.52 ± 1.01	6.25 ± 0.95
VA/P (mm/s)						
Young	3.70 ± 0.61	4.05 ± 0.57	3.64 ± 0.61	4.30 ± 0.72	4.35 ± 0.75	3.87 ± 0.63
Elderly	4.23 ± 0.72	4.40 ± 0.62	4.17 ± 0.66	5.19 ± 0.80	5.40 ± 0.82	4.99 ± 0.82
VM/L (mm/s)						
Young	2.52 ± 0.58	2.93 ± 0.74	2.43 ± 0.73	2.91 ± 0.71	3.19 ± 0.76	2.65 ± 0.63
Elderly	3.21 ± 0.38	3.31 ± 0.41	3.07 ± 0.39	3.63 ± 0.54	3.69 ± 0.54	3.44 ± 0.51

The table shows the analysis of postural parameters for CoPDISP = Center of Pressure displacement, CoPVEL = sway velocity of Center of Pressure, VA/P = anteroposterior sway velocity, VM/L = mediolateral sway velocity in both group (elderly and young) on three surfaces Firm, Foam and Firm textured Surface (FTS)

Separate four-way ANOVAs were applied to each dependent variable, and the type of surface, vision, age, and gender were identified as the experimental factors (Table 2).

**Table 2:** Measurement of postural control under different sensory conditions

Effects	CoPDISP	
	*F* value	*p-*value
Surface	41.89	< 0.0001
Vision	83.99	< 0.0001
Age	21.61	< 0.0001
Vision × Age	17.90	0.0001

Effects	CoPVEL	
	*F* value	*p*-value
Surface	28.40	< 0.0001
Vision	68.23	< 0.0001
Age	19.95	< 0.0001
Vision × Age	10.66	0.0023

Effects	VA/P	
	*F* value	*p-*value
Surface	17.63	< 0.0001
Vision	68.06	< 0.0001
Age	16.26	0.0003
Surface × Vision	4.46	0.0181
Vision × Age	18.82	0.0001

Effects	VM/L	
	*F* value	*p-*value
Surface	26.46	< 0.0001
Vision	37.02	< 0.0001
Age	18.32	0.0001
Surface × Age	3.57	0.0380

The table shows the significant effects on CoPDISP, CoPVEL, VA/P and VM/L of surfaces (Firm as control surfaces, Foam and FTS), vision (open and closed eyes), age (young and elderly) and the significant interactions. *P* < 0.05

The main effect of the surface was significant in all the four fitted models (*p* < 0.0001): the observed F values were 41.89, 28.40, 17.63 and 26.46 for CoP_DISP,_ CoP_VEL,_ V_A/P_ and V_M/L,_ respectively.

Vision showed a significant main effect on each response variable (*p* < 0.0001): the observed F values were 83.99, 68.23, 68.06 and 37.02 for CoP_DISP,_ CoP_VEL,_ V_A/P_ and V_M/L,_ respectively.

The main effect of age was significant in each of the four fitted models (*p* < 0.0001 for CoP_DISP,_ CoP_VEL_ and_,_ V_A/P_; *p* = 0.0003 for V_M/L_): the observed F values were 21.61, 19.95, 16.26 and 18.32 for CoP_DISP,_ CoP_VEL,_ V_A/P_ and V_M/L,_ respectively.

Nevertheless, the gender was never significant, either as a main effect or an interaction with other experimental factors.

When CoP_DISP_ was the response variable, the main effects of the surface were shown in Table 3. In details, FTS was more stable in comparison to the foam (estimated difference (e.d.)  – 11.53 mm, standard error (s.e.) 1.26) and the firm surfaces (e.d.  – 8.10 mm, s.e. 1.67), while no significant difference was detected between the firm and foam surfaces. Moreover, in the elderly group the instability was observed under both visual conditions (e.d. + 13.11 mm, s.e. 4.10), especially during eyes-closed condition (e.d. + 25.40 mm, s.e. 4.66).

**Table 3:** Estimated differences at multivariable analysis

Effect	CoPDISP		
	Estimated difference (mm)	s.e	*p-* value
Surface			
FTS – Foam	– 11.53	1.26	< 0.0001*
FTS – Firm	– 8.10	1.67	< 0.0001*
Firm—Foam	3.44	1.46	0.0596
Vision × Age			
OE Elderly—Young	13.11	4.10	< 0.0142*
CE Elderly—Young	25.40	4.66	< 0.0001*

	CoPVEL		
	Estimated difference (mm/s)	s.e	*p-* value
Effect			
Surface			
FTS – Foam	– 0.48	0.06	< 0.0001*
FTS – Firm	– 0.31	0.08	0.0010*
Firm—Foam	0.18	0.07	0.0298*
Vision × Age			
OE Elderly—Young	0.65	0.20	< 0.0132*
CE Elderly—Young	1.20	0.24	< 0.0001*

	VA/P		
Effect	Estimated difference (mm/s)	s.e	*p-*value
Surface x Vision			
OE FTS – Foam	– 0.32	0.07	0.0014*
OE FTS – Firm	– 0.06	0.07	0.9584
OE Firm—Foam	0.26	0.08	0.0300*
CE FTS – Foam	– 0.45	0.08	< 0.0001*
CE FTS – Firm	– 0.32	0.09	0.0095*
CE Firm—Foam	0.13	0.09	0.6604
Vision x Age			
OE Elderly—Young	0.42	0.18	0.1006
CE Elderly—Young	1.06	0.22	0.0001*

Effect	VM/L		
	Estimated difference (mm/s)	s.e	*p-* value
Vision			
OE—CE	– 0.34	0.06	< 0.0001*
Surface × Age			
Young FTS – Foam	– 0.51	0.07	< 0.0001*
Young FTS – Firm	– 0.17	0.08	0.2671
Young Firm—Foam	0.34	0.09	0.0041*
Elderly FTS – Foam	– 0.26	0.07	0.0146*
Elderly FTS – Firm	– 0.18	0.08	0.2247
Elderly Firm—Foam	0.08	0.09	0.9440

The table shows the differences of positive effects on CoP displacement, CoPVEL, VA/P and VM/L

*P < 0.05

When CoP_VEL_ were the response variable, the FTS was confirmed as the most effective surface in reducing instability, the estimated difference was  – 0.48 mm/s, (s.e. 0.06) and  – 0.31 mm/s (s.e. 0.08) compared to the foam and the firm surface, respectively (Table 3). As already observed in CoP_DISP_, the gap between elder and young people was more evident when subjects closed their eyes.

In addition, when CoP_DISP_ and CoP_VEL_ were the response variable, we detected a significant interaction between vision and age (Table 3).

When V_A/P_ was modeled, the significant interaction of surface x vision and vision × age were reported in Table 3. In detail, the difference between FTS and the foam was significantly higher in the eyes-closed condition than the eyes-open condition (e.d.  – 0.45 mm/s, s.e. 0.08 vs e.d.  – 0.32 mm/s, s.e. 0.07). Similarly, the data showed that the difference between FTS and the firm was negligible with eyes open, while becoming significant with eyes closed (e.d.  – 0.32 mm/s, s.e. 0.09). The difference between the firm and the foam was only significant in the eyes-open condition (e.d. 0.26 mm/s, s.e. 0.08). No significant difference was detected between the elderly and the young with eyes open, while this difference was significant in the eyes-closed condition (e.d. 1.06 mm/s, s.e. 0.22).

In V_M/L_ analysis (Table 2), a single significant interaction Surface × Age was detected. In Table 3, it is possible to note a constant worsening effect of the eyes-closed condition (e.d.  – 0.34 mm/s, s.e. 0.06). The difference between FTS and the foam was significant in both the elderly and young subjects, but the stabilizing effect was higher at the end (e.d.  – 0.26 mm/s, s.e. 0.07 *vs* e.d.  – 0.51 mm/s, s.e. 0.07). FTS and the firm showed similar results with eyes open and closed.

## Discussion

According to several investigations that demonstrated how a foam surface had poor performances resulting in the worst surface material for upright posture in elderly and young people (Chiang and Wu 1997; Perry et al. 2000; Nurse et al. 2005; Fransson et al. 2007), the data of this study suggest that FTS with no deformable spikes influence postural control system through tactile sensory inputs of the plantar foot, in both the elderly and the young people (Orth et al. 2013). These results seem to confirm previous evidence, despite using different surface materials (Palluel et al. 2008, 2009; Qiu et al. 2012). The characteristics of materials such as shape, contour and hardness could affect the degree of deformation of skin receptors (Orth et al. 2013) and consequently affect their stimulation to some degree (Hatton et al. 2011; Qiu et al. 2012).

Tests carried out by various studies have shown that height, texture, and spacing between spikes stimulate differently plantar surface, thus justifying controversial results found by various authors (Viseux et al. 2019). In a study, three textured surfaces with different spacing between protrusions (20-15-10 mm) have been investigated, finding an improvement in postural stability as distance between protrusions decreased, both with eyes open and closed (Watanabe et al. 1981). The height (3.5 mm) and distance between the spikes (17 mm) of the FTS used in this study could be optimal for achieving better plantar stimulation in both the young and the elderly people. Presumably, there was greater sensory discrimination by plantar mechanoreceptors and consequently more efficient neuromuscular activity (Forth and Layne 2007, 2008) which could justify the more consistent results compared to other studies. In fact, some authors have stated that if plantar skin is adequately stimulated, postural control improves probably through more afferent information from plantar mechanoreceptors to the Central Nervous System (Manjarrez et al. 2003; Menant et al. 2008; Viseux et al. 2018).

In addition, vision plays a crucial role in generating an internal model of body in space (Peterka 2002). In this study, the analyzed postural parameters increased significantly with eyes closed in comparison with eyes open, but according to Kenny et al. it was surprising that there was no significant interaction between surface and vision (Kenny et al. 2019b), except to V_A/P_. In fact, post-hoc analysis showed a significant reduction in V_A/P_ during static upright posture on FTS in comparison to the foam in the EO and EC conditions as shown in Table 3. On the other hand, in the comparison with the firm surface, a significant reduction was observed only with EC. These results may confirm hypothesis of previous studies about the beneficial effects of plantar sensory stimulation through a textured surface (Palazzo et al. 2014, 2019; Annino et al. 2015, 2018), especially in eyes-closed conditions (Qiu et al. 2012; Kenny et al. 2019b), and the negative effects of the foam surface on postural control (Teasdale et al. 1991).

The significant effects on CoP_DISP_, CoP_VEL_, V_A/P_,and V_M/L_ were also evident in the age analysis seem to confirm previous studies (Winter et al. 1996; Kitabayashi et al. 2011; Pasma et al. 2014) reporting a large body sway in elderly compared to young people due to a decrease in overall somatosensory function in over 60′s (Magnusson et al. 1990; Collins et al. 1995; Demura et al. 2008).

The significant two-interaction Vision x Age for CoP_DISP_, CoP_VEL_, and V_A/P_, showing a larger gap between the elder and younger people, especially in the EC condition. Instead, the significant two-interaction Surface × Age in V_M/L_ showed very poor performances on the foam surface, suggesting that FTS should be preferred especially in the elderly subjects. In line with the results of the present study, the velocity parameters in the ML direction were suggested to detect age-related differences in the permanent’s quality balance (Raymakers et al. 2005; Pasma et al. 2014). Differently from young adults where A/P movement plays a significant role (ankle strategy), in older adults, M/L movement (hip strategy) is prevalently adopted (Jančová 2008; Hatton et al. 2011). Considering that M/L balance control strategy, can cause accidents in falls or severe injury in elderly people (Eibling 2018), these results seem to intervene in this direction helping hip strategy. Differently from A/P direction where balance strategy is facilitated by the correction of stepping forward or backward (Winter et al. 1996), in M/L balance strategy unsupported lower limb is on opposite side of direction of fall and, in this case, it would be difficult to recover balance quickly (Rogers and Mille 2003). This improvement could be due both to stimulate tactile sensory inputs of the plantar foot by the semi-circular protrusions and a different multiple-link strategy between the surfaces. It is well know that four kinds of cutaneous mechanoreceptors that deliver important feedback about the environment and innervate glabrous skin (Patel et al. 2008): the rapidly adapting Meissner’s corpuscle (MC) and Pacinian corpuscle (PC), the slowly adapting Merkel disk and Ruffini’s ending (Patel et al. 2008). Each of these neuron types responds to cutaneous motion and deformation in a different way (Johnson 2001). Also, both Pacinian and Meissner’s receptors have been associated with declines in touch thresholds. Especially the cutaneous slowly adapting type 1 (SA1) has proven to be the main responsible for shape and roughness perception by responding to a sustained skin deformation with a prolonged discharge. In fact, it has been demonstrated in previous studies that the perception of texture depends on the distributed statistical properties of a surface or material (Kenny et al. 2019a) and on a measure of the neural response SA1 which is extracted from the central neurons with simple excitatory and inhibitory subfields (Johnson 2001).

Also, this study did not show a significant difference between males and females in all the analyzed postural parameters agreeing to previous studies (Røgind et al. 2003; Demura et al. 2008). Nakamura et al. did not find a significant difference between genders in young subjects, but only in elderly subjects where males were more unstable than females, suggesting that the differences in height could explain the result being the tallest male (Nakamura et al. 2001). In our study, there were differences in average height between the males and females in the young and elderly groups and the analyzed data suggested that these differences did not influence postural stability. Instead, in a study by Overstall et al., males showed higher stability than females (Overstall et al. 1978). More in detail, Kim et al. showed a difference between sexes and age-related balance, especially in mediolateral direction (Kim et al. 2010). Despite the results of this study where postural control is not influenced by sex, further and depth investigation should be needed in future.

In conclusion, the most realistic hypothesis presently is that a textured firm surface improves postural control in the all groups (significant main effect of the surface) and as a result should be especially interesting in older subjects known to exhibit deficits in postural stability. A firm-textured surface stimulated better sensory plantar receptors in all the groups. The characteristics of the textured material, such as hardness, height, and spacing between the protrusions could be essential to achieve an optimal sensory stimulation and consequently improve better postural control. In addition, it is also presumable that the surface x age interaction with a larger sample of elderly subjects and/or with more trials, could have reached significance. The difference between both groups on the foam surface is considerably less compared to the other surfaces. Further gait analysis with surface electromyography (EMGs) studies using non-deformable stimulating surface needed to observe the influence of plantar sensory inputs and joint strategies on neuromuscular control.
